# Measuring Coping Strategies in Daily Life: A Systematic Review of Experience Sampling Methodology and Daily Diary Studies

**DOI:** 10.1002/smi.70223

**Published:** 2026-09-13

**Authors:** Petra Mikolič Brence, Sara Bugarinović, Gaja Zager Kocjan

**Affiliations:** ^1^ Department of Psychology Faculty of Arts University of Ljubljana Ljubljana Slovenia; ^2^ Mental Health Centre National Institute of Public Health Ljubljana Slovenia

**Keywords:** coping strategies, daily diary, daily stressors, experience sampling method (ESM), systematic review

## Abstract

Advances in daily diary methods and experience sampling method (ESM) have improved the study of coping strategies in daily life and their role in shaping health and well‐being. In this review, we examine study designs, measurement approaches, and analytical practices used to investigate coping in natural contexts. We performed a systematic review of studies published before 5 December 2025 that used daily diary or ESM to measure coping strategies over multiple days or moments. Studies were examined with regard to sampling schemes, assessment frequency and duration, measurement of coping strategies, incorporation of stressor appraisals, and analytic techniques used to model coping processes. Fifty‐five studies met the inclusion criteria. Results indicated that 80% employed end‐of‐day diary designs, generally lasting 1–3 weeks, whereas higher‐frequency ESM protocols were less common and ranged 2–14 days. Coping strategies were often assessed using abbreviated or single‐item measures, frequently adapted from established questionnaires. Many studies incorporated appraisals such as perceived stressor intensity or controllability, enabling tests of coping flexibility. Multilevel modelling was the dominant analytic approach, allowing researchers to distinguish within‐person dynamics from between‐person differences. However, analyses were predominantly concurrent, and temporally ordered models remained comparatively rare. Overall, the literature demonstrates substantial progress in capturing coping in everyday contexts, yet heterogeneity in measurement and limited use of temporal modelling constrain cumulative knowledge about the temporal links between coping and psychological and physiological health outcomes. Future research would benefit from greater alignment between theoretical assumptions, assessment strategies, and analytic methods.

## 1 Introduction

Stress is an inevitable part of daily life, and understanding how individuals cope with challenges has been a longstanding focus of psychological research (Litt et al. 2011). Coping refers to the emotional, cognitive, and behavioural efforts to manage, reduce, or tolerate internal and external demands (Lazarus and Folkman 1984). Within biopsychosocial models of stress, coping is viewed as a key process linking stress exposure to psychological and physical health outcomes (Litt et al. 2011). Over the past decades, researchers have described hundreds of distinct coping strategies (Skinner et al. 2003), reflecting the complexity and diversity of stress responses. Despite this conceptual richness, much of the empirical research on coping has been limited to static, retrospective self‐report measures that typically assess individuals' general coping tendencies rather than capturing how individuals adjust their strategies across situations and over time (Litt et al. 2011; Todd et al. 2004). As psychological science increasingly emphasises dynamic, context‐sensitive models of behaviour (Revelle and Wilt 2021), the coping research is shifting towards more ecologically valid approaches that can capture within‐person variability and real‐time coping behaviour (Litt et al. 2011; Trudel‐Fitzgerald et al. 2024). Technological and methodological advances have made such approaches more feasible, particularly through the use of intensive longitudinal designs. Across the literature, these intensive longitudinal approaches are referred to using a range of partially overlapping terms, including ecological momentary assessment (EMA), experience sampling method (ESM), ambulatory assessment, and daily diary methods. Although these terms differ somewhat in emphasis, they all refer to approaches designed to capture psychological processes in everyday life (Bolger et al. 2003; Conner and Lehman 2012; Trull and Ebner‐Priemer 2014).

Despite growing interest in these methods, studies vary considerably in their sampling strategies, assessment schedules, operationalizations of coping, and analytical approaches. These methodological choices can substantially influence the coping processes that are observed, the conclusions drawn from individual studies, and the comparability of findings across the literature, making it difficult to evaluate the strengths and limitations of existing approaches and to identify methodological priorities for future research. To date, however, no systematic review has focused specifically on how coping strategies are measured using intensive longitudinal designs. The present review addresses this gap by providing the first systematic synthesis of studies assessing coping with everyday stress using ESM and daily diary methods, with the aim of clarifying current methodological practices, identifying areas of convergence, inconsistency, and gaps, and informing future methodological decisions in daily‐life coping research.

Although coping was initially conceptualised as a dynamic and transactional process (Lazarus and Folkman 1984), it has often been studied as a relatively stable trait. Many studies assess individuals' general coping tendencies without specifying a particular time frame or context—for example, how people typically respond to stressful events (e.g., the COPE Inventory; Carver et al. 1989)—or ask participants to reflect on a single, supposedly representative stressful event (e.g., Coping Responses Inventory; Billings and Moos 1984). Several factors explain the persistence of these static, retrospective approaches. First, such questionnaires are easy to administer, require fewer resources, and allow for efficient data collection from large samples. Second, many theoretical models assume coping styles are consistent across situations (e.g., Billings and Moos 1984), reinforcing the use of trait‐based assessments. Third, early technological and logistical limitations made it difficult to collect intensive, repeated data in daily life contexts (e.g., Stone et al. 1995).

However, the landscape of psychological research has changed. Advances in mobile technologies, digital assessment platforms, and advanced statistical methods have made it increasingly feasible to capture coping processes in everyday life through intensive longitudinal designs. ESM typically involves repeated in‐the‐moment assessments administered several times per day, whereas daily diary studies usually rely on one end‐of‐day report summarising daily experiences. Together with other forms of repeated real‐time assessment, such as ambulatory assessment incorporating passive behavioural or physiological monitoring, these methods can be situated within the broader framework of ecological momentary assessment (EMA; Horstmann 2021). By capturing experiences close to their occurrence, these approaches substantially reduce retrospective bias and provide rich, time‐sensitive data on stress, emotions, and coping in naturalistic contexts (Bolger et al. 2003; Conner and Lehman 2012; Larson and Csikszentmihalyi 2014).

Even with their clear advantages, intensive longitudinal methods remain demanding to implement. They require frequent data collection, often over multiple days or weeks, which can increase participant burden and lead to higher dropout rates (Scollon et al. 2003). These studies also require a lot of time, staff, and technical resources, such as setting up reminders and managing the data. As a result, they are often more expensive and time‐consuming than traditional approaches (Conner and Lehman 2012). Moreover, analysing intensive longitudinal data requires specialised statistical techniques that account for the nested structure of repeated observations within individuals. Methods such as multilevel (hierarchical) modelling, time‐lagged analyses, and multilevel mediation and moderation allow researchers to disentangle within‐person variability from between‐person differences and to examine how coping strategies relate to outcomes both concurrently and over time (Bolger and Laurenceau 2013; Curran and Bauer 2011). These approaches are critical for capturing the dynamic and context‐dependent nature of coping processes, offering insights that static, cross‐sectional designs cannot provide.

Nonetheless, there is a growing recognition that dynamic, context‐sensitive assessments are essential for advancing theoretical models of coping and informing interventions aimed at enhancing adaptive coping (Cheng et al. 2014). For example, a recent daily diary study found that using emotion‐focused coping in uncontrollable situations was linked to lower depressed mood, while using the same strategy in controllable situations was associated with worse mood outcomes (Leslie‐Miller et al. 2025). These findings are consistent with the concept of coping flexibility, defined as the capacity to modify or switch coping strategies based on situational demands—a capacity that has been shown to predict better psychological adjustment to stressful life changes (Cheng et al. 2014). These perspectives emphasise the importance of assessing coping in context, rather than relying solely on generalised trait measures. Importantly, studies have shown only low to moderate correlations between global, retrospective reports and momentary assessments of coping (Todd et al. 2004), suggesting that traditional measures and real‐time assessments capture different facets of coping. Whereas the former reflect broad tendencies or self‐perceptions, the latter provide a more context‐specific and ecologically valid view of coping as a dynamic process.

### 1.1 This Review

While a recent review by Trudel‐Fitzgerald et al. (2024) acknowledged the increasing use of intensive longitudinal approaches, its primary focus was to map the conceptual and measurement overlaps between coping and emotion regulation frameworks. To date, however, no systematic review has specifically synthesised how coping strategies have been measured using these intensive longitudinal designs. The present review therefore complements existing work by providing the first systematic overview of current methodological practices in daily‐life coping research.

Specifically, this review synthetises studies that used ESM or daily diaries to assess coping with everyday stress in non‐clinical populations. We aim to: (I) examine key study design characteristics, including sampling schemes, timeframes, frequency, and duration of assessments, (II) review the measurement of coping strategies, including instruments and items used, response formats, and how stressor characteristics (e.g., perceived intensity, controllability) were incorporated, and (III) summarise the analytical approaches applied to intensive longitudinal coping data. By bringing these findings together, this review aims to identify common patterns, methodological differences, and gaps in the current literature on coping, and to critically evaluate these approaches for advancing research on the dynamic links between coping, stress, and health in everyday life. In this way, the review provides a methodological overview of the field that may inform the design of future intensive longitudinal studies of coping and facilitate comparisons across studies.

## 2 Method

### 2.1 Search Strategy and Eligibility Criteria

We searched PsycINFO, MEDLINE, and Scopus for English‐language articles using subject headings (e.g., thesaurus terms and MeSH headings) and free‐text title, abstract and, keyword terms related to coping and intensive longitudinal methods. Coping terms included headings such as Coping Behaviour, Coping Style, Stress and Coping Measures, and Coping Skills, as well as truncated text words (e.g., coping*, cope, coped, copes). Method terms included headings such as Diary Measure and Ecological Momentary Assessment, as well as text words (daily diar*, experience sampling, ambulatory assessment, EMA, ESM). Where possible, searches were limited to peer‐reviewed records. The search was conducted on December 5, 2025. We also reviewed reference lists of included studies and relevant literature found during manuscript preparation.

To be eligible, studies had to be peer‐reviewed, published in English, use daily diary or ESM methods to assess coping strategies at least once per day, and clearly measure coping as intentional efforts to manage stress. Studies using daily reconstruction method (DRM; Kahneman et al. 2004) to assess coping across retrospectively reconstructed episodes of the previous day were outside the scope of the review, as DRM represents a methodologically distinct approach. Studies were also required to include self‐reported coping strategies (rather than related constructs such as coping efficacy or proxy reports) and to allow participants to report coping with any naturally occurring stressor (i.e., not restricted to discrimination, chronic pain, or work stress). We excluded studies focussing on clinical populations or intervention studies, those lacking clear measurement details (e.g., number of items or instruments not reported), and those assessing behaviours such as alcohol use without explicitly linking them to coping motives. When multiple publications were based on the same dataset, only one article was included in the review. Preference was given to the publication providing the most comprehensive methodological information, particularly regarding the assessment of coping. If multiple publications were comparable in scope and methodological detail, the earliest published article was retained.

### 2.2 Screening and Inclusion

**FIGURE 1:**
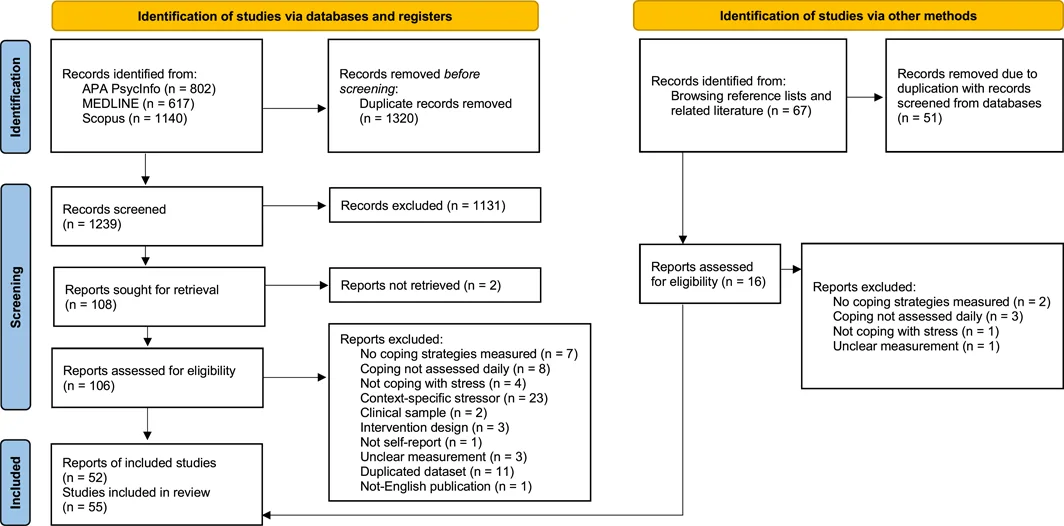
PRISMA flow diagram.

The selection process is shown in Figure 1. We identified 2559 records. After removing duplicates, 1239 were screened by title and abstract. PMB and SB independently screened titles and abstracts, followed by full text assessment of potentially eligible articles. Agreement was 93.3% at the title/abstract stage and 81.1% at the full‐text stage. Discrepancies were resolved through discussion.

After full‐text review, 43 reports met inclusion criteria. Manual reference and related literature checks identified 16 additional records, of which nine were eligible. In total, 52 reports were included, comprising 55 studies.

## 3 Results

Out of 55 included studies, 37 (67.3%) were published from 2016 to 2025, 11 (20.0%) from 2006 to 2015, and 7 (12.7%) from 1995 to 2005. The list of the studies is shown in Table 1.

**TABLE 1:** Studies included in review.

Study	Sample and study duration	Coping measures	Other stress measures	Analytical approaches
**Measuring coping strategies once per day**				
Assessing coping with (the most) stressful event				
Aldridge and Roesch (2008)	67 adolescents (*M*_*age*_ = 15.5, 60% girls); 5 days	8 items (1 per strategy): Planning, direct problem solving, positive thinking, acceptance, distancing actions, religious coping, support for actions, humour. Instruments: CCSC, HICUPS; 4‐point scale.	Description of the most stressful event; event stressfulness	Linear MLM estimating same‐day associations between daily coping and affect, controlling for L1 stress severity and L2 age/gender
Blaxton and Bergeman (2017)	371 older adults (*M*_*age*_ = 67.41, 64% women); 56 days (burst design)	7 items (1 per strategy): Acceptance (resituating); social support, direct action, situation redefinition (meaning‐making); distraction, catharsis, relaxation (dispelling). Instrument: DCI (modified for older population); binary response.	Event stressfulness; event controllability	Linear MLM with WP/BP decomposition of daily coping and appraisal predicting same‐day affect; tested WP coping × appraisal and cross‐level age × coping × appraisal; included time trends
David and Suls (1999)	95 men (*M*_*age*_ = 42.3); 8 days	8 items (1 per strategy): Distraction, situation redefinition, direct action, catharsis, acceptance, seeking social support, relaxation, religion. Instrument: DCI; binary response.	Description of the most stressful event (daily, past, or anticipated); event controllability; event undesirability	Logistic MLM estimating same‐day associations between daily appraisals and coping; L2 traits predicted mean coping and moderated L1 slopes
Donald et al. (2016)	143 university students and staff (*M*_*age*_ = 33.7, 76% women); 20 days (four weekly blocks)	1 item: avoidance coping. Instrument: Adapted from the behavioural disengagement subscale of the brief COPE; 5‐point scale.	Present‐moment awareness; threat appraisal; perceived self‐efficacy; values‐consistent responding	Linear MLM with WP/BP decomposition estimating same‐day effects of present‐moment awareness on avoidance coping (controlling L1 threat appraisal/NA); lagged (next‐day) models with autoregressive control
Dunkley et al. (2014)	196 community adults (*M*_*age*_ = 40.94, 66% women); 14 days	24 items (4 per strategy): Denial, behavioural disengagement, mental disengagement (avoidant coping); active coping, planning (problem‐focused coping); positive reinterpretation and growth. Instrument: COPE; 4‐point scale.	Description of the most stressful event; event unpleasantness; event duration; event stressfulness; event controllability; perceived criticism	Multilevel SEM: Same‐day WP indirect pathways among daily appraisals → coping → affect; prospective BP indirect effects from T1 traits to T2 aggregate daily processes (6‐month follow‐up)
Ferrer et al. (2021)	122 adults (*M*_*age*_ = 42.7, 65.6% women); 10 days	10 items (2–3 per coping style): Problem‐focused coping, emotion‐focused coping, support‐seeking, refusal. Instrument: MoCope coping questionnaire; 5‐point scale	Daily stressors checklist; discomfort caused by stressors; threat, challenge and loss appraisals; event controllability	Linear MLM estimating same‐day associations between appraisal and coping; L2 traits predicted mean coping (and in some models moderated slopes)
Finkelstein‐Fox et al. (2022)	261 undergraduate students (*M*_*age*_ = 18.9, 74% women); 11 days	14 items (1 per strategy): Active coping, planning, instrumental support (problem‐focused); positive reframing, acceptance, religious coping (meaning‐focused); behavioural disengagement, denial, substance use (avoidance); emotional support, venting, humour, self‐blame, self‐distraction. Instrument: Brief COPE; 4‐point scale.	Daily stressors checklist; event stressfulness; event controllability	Multilevel mediation estimating same‐day WP indirect effects of daily appraisals on affect/somatic symptoms via coping; cross‐level moderation (L2 victimization × L1 stressor type); time covariate included
Finkelstein‐Fox et al. (2019)	157 undergraduate students (*M*_*age*_ = 17.81, 79% women); 7 days	14 items (1 per strategy): Active coping, positive reappraisal, acceptance, behavioural disengagement, self‐blame; planning, instrumental support, religious coping, denial, substance use, emotional support, venting, humour, self‐distraction. Instrument: Brief COPE; 8‐point scale.	Daily stressors checklist; event stressfulness; event controllability	2‐1‐1 multilevel mediation: L2 mindfulness → L1 affect via aggregated daily coping, appraisal, and fit indices (BP mediation using person‐level aggregates); time covariate included
Friedman‐Wheeler et al. (2019)	71 adults (*Mdn*_*age*_ = 27, 68% women); 7 days	13 items (1 per strategy): Eat/drink/smoke, blame the other person, ruminate, avoid reminders, isolate, address the problem directly, talk to someone, do nothing, acceptance, cognitive restructuring, seek distraction, religion/spirituality, exercise. Instrument: Self‐developed; binary response.	Description of the most stressful event	Logistic MLM estimating same‐day L1 coping as a function of L2 mood‐regulation expectancies; stratified by stressor context (interpersonal vs. achievement)
Galla and Wood (2015)	129 ninth‐grade adolescents (*M*_*age*_ = 14.7, 59% girls); 14 days	4 items (4 per strategy): Cognitive reappraisal. Instruments: RSQ, ERQ; 4‐point scale.	Daily stressors checklist; daily stress severity	Linear and Poisson MLMs estimating same‐day daily associations among stress, mindlessness, and coping, with L2 self‐control predicting mean levels; indirect pathways involving daily coping
Gunthert et al. (1999)	197 students (approx. 18–20 years old, 68% women); 14 days	13 items (1 per strategy): Distraction, situation redefinition, direct action, catharsis, acceptance, seeking social support, relaxation, religion, self‐blame, hostile reaction, wishful thinking, information seeking, humour. Instrument: Based on DCI; binary response	Description of the most stressful event; event controllability; event desirability; event result; event stressfulness; perceived self‐efficacy	Linear MLM estimating same‐day daily associations among appraisals, coping, and negative affect; L2 neuroticism predicted mean affect and moderated L1 slopes
Hoggard et al. (2012)	35 african american college students (*M*_*age*_ = 19.08, 74% women); 20 days	13 items (1–4 per strategy): Confrontive, seeking social support, planful problem solving (problem‐focused), avoidance, internal, rumination (emotion‐focused). Instrument: WCCL and three self‐developed items; 4‐point scale.	Description of the stressful event; coping demands; event stressfulness; coping efficacy	MLMs estimating same‐day differences in coping strategy use across racial versus nonracial stressors using WP comparisons
Iida et al. (2017)	321 law graduates preparing for the bar exam (*M*_*age*_ = 29.8, 55% women); 35 days	12 items (1–2 per strategy): Acceptance, active coping, alcohol/drug disengagement, emotional support seeking, mental disengagement, planning, positive reinterpretation, practical support seeking, religion, venting. Instrument: COPE; binary response.	Listing of the most stressful aspect of their day; whether it was bar Em‐related	Linear MLM estimating lagged (previous‐day → next‐morning) effects of coping on anxiety, with WP/BP decomposition of coping and autoregressive control; tested stress‐phase moderation (high vs. low stress periods)
Kleinman and Quinn (2025)	130 undergraduate students (*M*_*age*_ = 19.15, 59% women); 14 days	28 items (2 per strategy): Active coping, planning, instrumental support (problem‐focused); positive reframing, emotional support, acceptance, religious coping; humour (emotion‐focused); behavioural disengagement, denial, substance use; venting, self‐blame, self‐distraction. Instrument: Brief COPE; 4‐point scale	Goal clarity; regulation success	MLM with WP/BP decomposition estimating same‐day effects of daily goal clarity and coping on regulation success; tested WP goal clarity × coping interactions and BP main effects
Leslie‐Miller et al. (2025)	75 college students (*M*_*age*_ = 18.51; 65% women); 7 days	28 items (2 per strategy): Active coping, planning, instrumental support (problem‐focused); positive reframing, emotional support, acceptance, religious coping; humour (emotion‐focused); behavioural disengagement, denial, substance use; venting, self‐blame, self‐distraction. Instrument: Brief COPE; 4‐point scale.	Event stressfulness; event controllability	GLMM estimating same‐day associations between coping and depressed mood; tested coping × controllability × stress interactions using person‐centred predictors
Monroy et al. (2021)—Study 1	110 community latinx adults (*M*_*age*_ = 43.25, 62% women); 7 days	6 items (1 per strategy): Acceptance, active coping, emotional support, humour, positive reframing, planning. Instrument: Brief COPE; 8‐point scale.	Description of the most stressful event; event stressfulness	Multilevel mediation estimating same‐day WP indirect associations between adaptive coping and well‐being via positive emotions (person‐mean centred); L2 covariates included
Monroy et al. (2021)—Study 2	374 undergraduate students (*M*_*age*_ = 20.56, 76% women); 14 days	6 items (1 per strategy): Acceptance, active coping, emotional support, humour, positive reframing, planning. Instrument: Brief COPE; 8‐point scale.	Description of the most stressful event; event stressfulness	Replication of the WP multilevel mediation used in study 1; BP lagged mediation: Average coping (days 1–7) → later well‐being (T2) via average positive emotionality (days 8–14)
Park et al. (2004a)	137 students (*M*_*age*_ = 18.72, 74% women); 28 days	12 items (4 per coping style): Emotion‐focused coping, avoidance coping, problem‐focused coping. Instruments: Brief COPE, CTEAS; 4‐point scale.	Description of the most stressful event; event stressfulness	Poisson and linear MLM estimating same‐day associations among stress, coping, affect, and alcohol use; L2 moderators of L1 slopes
Park et al. (2004b)	190 students (*M*_*age*_ = 18.65, 75% women); 28 days	14 items (1–3 per coping style): Active problem solving, social support/emotion‐focused coping, acceptance/restraint, disengagement/denial, processing emotions, expressing emotions. Instruments: COPE, CTEAS; 4‐point scale.	Description of the most stressful event; event stressfulness; event controllability	Linear MLM estimating same‐day coping–mood associations and WP coping × controllability (goodness‐of‐fit) interactions
Roesch et al. (2009)	89 low‐income minority adolescents (*M*_*age*_ = 15.4, 49% girls); 3, 4, or 5 days (3 groups)	8 items (1 per strategy): Support for actions, humour, problem‐solving, planning, positive thinking, acceptance, religious coping, distraction. Instruments: CCSC, HICUPS; 4‐point scale.	Description of a stressful event; event stressfulness; event attributions	MLM estimating same‐day indirect pathways from daily attributions to affect via coping
Roesch et al. (2010a)	366 college students (*M*_*age*_ = 20.14, 69% women); 5 days	28 items (2 per strategy): Cognitive decision making, direct problem solving, seeking understanding, positive cognitive restructuring, expressing feelings, humour, religious coping, physical release of emotions, distracting actions, avoidant actions, cognitive actions, cognitive avoidance, problem‐focused support, emotion‐focused support, acceptance. Instruments: CCSC, HICUPS, RSQ; 4‐point scale.	Description of a stressful event	Multilevel factor analysis and MSEM separating BP and WP coping components. Structural paths from WP/BP latent coping factors to daily affect
Roesch et al. (2010b)	126 low income minority adolescent groups (*M*_*age*_ = 15.5, 48% girls); 5 days	8 items (1 per strategy): Support for actions, humour, problem‐solving, planning, positive thinking, acceptance, religious coping, distraction. Instruments: CCSC, HICUPS; 4‐point scale.	Description of the most stressful event; event stressfulness	Linear MLM predicting daily coping from BP hope components; focus on BP predictors of mean coping
Santiago et al. (2016)	58 adolescents (*M*_*age*_ = 13.31, 47% girls); 7 days	10 items (3–7 per coping style): Engagement coping, disengagement coping. Instrument: RSQ; 4‐point scale.	Daily stressors checklist; event stressfulness; involuntary responses to stress	Linear MLM estimating same‐day associations between stress and coping; L2 cultural variables predicting of mean levels and moderating L1 slopes
Sasaki and Kim (2011)	77 undergraduate students; 7 days	11 items (2–5 per strategy): Religious coping, adjustment of the self, acceptance, social coping. Instrument: Brief COPE and self‐developed items; 5‐point scale	None	Linear MLM estimating daily associations between religious coping and outcomes; disentangled WP versus BP religious coping; cross‐level moderation by culture tested
Shermeyer et al. (2019)	74 college students (*M*_*age*_ = 21.0, 85% women); 7 days	16 items (4 per coping style): Problem‐focused engagement, emotion‐focused engagement, problem‐focused disengagement, emotion‐focused disengagement. Instrument: CSI; 5‐point scale.	Description of the most stressful event; event stressfulness	Linear MLM testing same‐day WP associations between daily coping and mood/quality of life; person‐centred L1 predictors; L2 demographics predicted intercepts
Stone et al. (1995)	79 men (*M*_*age*_ = 43.2); approx. 115 days	8 items (1 per strategy): Distraction, situation redefinition, direct action, catharsis, acceptance, seeking social support, relaxation, religion. Instrument: DCI; binary response.	Description of the most stressful event (daily, past, or anticipated); event controllability; event undesirability; lifestyle change due to event; event anticipation; event meaningfulness; event chronicity; event stressfulness	WP (fixed‐effects) regression of same‐day coping → end‐of‐day mood; supplementary models adjusting for prior‐day mood
Todd et al. (2004)	93 community residents (*M*_*age*_ = 37.69, 54% women); 30 days	16 items (1 per strategy): Direct action, social support, relaxation, acceptance, restructuring, emotional expression, distraction, religion, avoidance, benefits, decision‐making/problem‐solving, comparison, emotional processing, perspective, prior successes, pleasurable activities. Instrument: DCI; 3‐point scale.	Positive and negative events checklist; event stressfulness	Multilevel analyses comparing daily coping reports with global and time‐limited retrospective reports; primarily descriptive concordance models rather than WP process MLM
Wu and Buchanan (2019)	30 undergraduate students (*M*_*age*_ = 18.67, 86% women); 14 days	Open‐ended question to report up to 4 coping strategies, then rated on: emotion suppression, cognitive reappraisal, problem solving. No formal instrument; 7‐point scale.	Description of three bothersome events, thoughts, or issues	Multilevel path models testing same‐day and lagged (*t* → *t* + 1 → *t* + 2) associations in which daily state mindfulness predicted coping and perceived stress, which in turn predicted vitality
Assessing coping with each stressful event separately				
Neupert and Bellingtier (2019)	107 younger adults (*M*_*age*_ = 19.44, 49% women) and 116 older adults (*M*_*age*_ = 64.71, 61% women); 8 days	15 items (2–5 per strategy): Problem analysis, plan rehearsal, stagnant deliberation, outcome fantasy. Instrument: MMAP; 5‐point scale.	Daily stressors checklist; daily stressor forecasting	Lagged MLM testing previous‐day forecasts and anticipatory coping in relation to next‐day stressor exposure and affective reactivity; age tested as an L2 moderator
Neupert et al. (2016)	43 older adults (*M*_*age*_ = 74.65, 91% women); 8 days	15 items (2–5 per strategy): Problem analysis, plan rehearsal, stagnant deliberation, outcome fantasy. Instrument: MMAP; 5‐point scale.	Daily stressors checklist; daily stressor forecasting	Lagged MLM testing WP fluctuations in anticipatory coping predicting next‐day health, affect, and memory outcomes
Assessing coping with overall stress/stressful experiences				
Galán et al. (2025)	371 ethnoracially minoritised adolescents (*M*_*age*_ = 14.47, 52% girls); 30 days (2 waves of 15 days)	10 items (2–4 per strategy): Problem solving, seeking social support, avoidance coping. Instrument: Short form of RSQ; 4‐point scale.	Exposure to racial‐ethnic discrimination	Linear MLM estimating same‐day associations between racial–ethnic socialisation, daily discrimination, proactive coping, and well‐being; lagged (next‐day) moderated mediation tested with prior‐day adjustment
Hulin et al. (2024)	55 employees (*M*_*age*_ = 34.2, 47% women); 15 working days	30 items total (4 per strategy); 2 per coping style randomly selected at each prompt: Active coping, planning (problem‐focused coping); focus on and venting of emotions (emotion‐focused coping); seeking emotional and instrumental support (social support). Instrument: COPE; 4‐point scale.	Threat appraisals; challenge appraisals	Linear MLM estimating same‐day associations between daily stress appraisals and coping strategies, using person‐centred L1 predictors and L2 covariates
Eckland and Berenbaum (2021)	211 undergraduate students (*M*_*age*_ = 19.4, 60% women); 7 days	2 items: Active coping. Instrument: COPE; 7‐point scale.	None reported	MLM estimating same‐day associations between daily emotional awareness and coping, with L2 emotional awareness predicting average daily coping
Lu and Shi (2024)	69 university students (*M*_*age*_ = 19.76, 24% women); 30 days	3 items: Emotion‐focused coping style. Instrument: Three items from CISS; 4‐point scale.	Daily academic stressors; daily perceived global stress	Linear MLM estimating same‐day effects of emotion‐focused coping on negative affect; L2 outcome‐oriented resilience predicting mean NA and moderating L1 slopes
Massey et al. (2009)	89 adolescents (*M*_*age*_ = 15.8; 74% girls); 21 days	9 items (1 per strategy): Acceptance, catastrophizing, other blame, positive reappraisal, positive refocus, putting into perspective, refocus on planning, rumination, self‐blame. Instrument: CERQ, 5‐point scale.	Level of daily frustration across 4 goal domains; coping efficacy	Logistic MLM testing same‐day and next‐day (lagged) associations of daily frustration, cognitive coping, and coping efficacy with headache occurrence
Modecki et al. (2022)	115 adolescents (*M*_*age*_ = 14.57, 73% girls); 7 days	3 items (1 per strategy): Self‐distraction, information seeking, emotional support. Instrument: Self‐developed; 5‐point scale.	None	MLM testing WP effects of online coping on subsequent emotion change, including curvilinear associations; prior affect entered as a covariate
Nguyen‐Feng et al. (2017)	260 undergraduate students (*M*_*age*_ = 21, 74% women); 14 days	8 items (2 per strategy): Self‐criticism, social withdrawal, wishful thinking, perceived present control. Instrument: CSI; binary response.	Daily stressors checklist; event controllability	SEM testing longitudinal mediation from baseline trauma exposure to later distress via aggregated daily avoidant coping and perceived control; models also estimated with L2 covariates
Sladek et al. (2017)	122 early adolescent girls (*M*_*age*_ = 12.39); 4 days	9 items (9 per coping style): Voluntary engagement coping. Instrument: RSQ; 4‐point scale.	Diurnal cortisol	Growth‐curve MLM with WP/BP coping; day‐level coping → next‐morning cortisol; BP coping × chronic stress predicting average diurnal parameters
Sutherland Charvis et al. (2023)	55 college students (*M*_*age*_ = 23.31, 85% women); 21 days	15 items (1–3 per strategy): Avoidance, relaxation, counselling, exercise, connection, media, prayer, other. Based on the centres for disease control and prevention website; binary response.	Stress level; feeling of being overwhelmed	GLMMs estimating same‐day WP associations between daily stress and coping; BP associations between average stress and coping
Valiente et al. (2015)	155 children (*M*_*age*_ = 9.14 at T1, 11.15 at T2, 13.41 at T3, 15.32 at T4; 76%–97% girls); burst design; 14 days per burst	9 items (1 per strategy): Cried, was very emotional (emotional coping); tried to ignore the problem, told myself there was not a problem, tried to stay away (avoidance coping); asked someone for help, talked to friends/family (support seeking); took action, cognitive restructuring. Based on prior diary protocol; binary response.	None reported	Longitudinal SEM (latent growth curve) using aggregated diary coping to model interindividual stability and mean‐level change across waves; coping examined at the BP developmental level
Ward et al. (2023)	251 undergraduate students (*M*_*age*_ = 20.07, 63% women); 12 days	4 items (2 per strategy): Positive reinterpretation, proactive coping. Instrument: Adapted COPE items; 7‐point scale.	Distressed emotions; COVID‐19 stress	MLM estimating WP same‐day associations between daily meaning and coping; lagged models testing bidirectional links between meaning and coping, controlling prior‐day levels
Weinstein et al. (2009)	70 students (*M*_*age*_ = 20, 83% women); 7 days	24 items (4 per coping style): Denial, behavioural disengagement, mental disengagement (avoidant coping); active coping, acceptance, positive reinterpretation and growth (approach coping). Instrument: COPE; 4‐point scale.	Listing of the most stressful event of the day; event stressfulness; daily stress level	Multilevel mediation estimating same‐day WP indirect effects of mindfulness on well‐being via stress appraisals and coping; models adjusted for day of week
Yap et al. (2021) –Study 1	191 young adults (*M*_*age*_ = 22.55, 67% women); 12 days	16 items (2–4 per strategy): Active coping, planning (problem‐focused coping); mental disengagement (avoidance‐oriented coping); emotional processing, emotional expression (emotional‐approach coping). Instruments: COPE, EACS; 4‐point scale.	Stress level	MLM estimating lagged (evening → next‐day) associations (WP stress × coping interactions predicting subsequent sleep); BP average coping tested as a cross‐level moderator; autoregressive controls for prior sleep
Yap et al. (2021)—Study 2	135 young adults (*M*_*age*_ = 24.76, 76% women); 7 days	20 items (4 per strategy): Active coping, planning (problem‐focused coping); mental disengagement (avoidance‐oriented coping); emotional processing, emotional expression (emotional‐approach coping). Instruments: COPE, EACS; 7‐point scale.	Stress level	MLM estimating lagged (evening → next‐day) associations (WP stress × coping interactions predicting subsequent sleep); BP average coping tested as a cross‐level moderator; autoregressive controls for prior sleep
**Measuring coping strategies multiple times per day**				
Assessing coping with (the most) stressful event				
Hammond et al. (2025)	240 community adults (*M*_*age*_ = 45.42, 71% women); 14 days (4 signals/day)	6 items (2 per coping style): Planning, active coping (problem‐focused), emotional support, instrumental support (support‐seeking), positive reframing, self‐distraction (cognitive coping). Instrument: Adaptation of brief COPE; 10‐point scale.	Coping flexibility	BP regression and path analysis estimating baseline optimism predicting change in depressive symptoms, with aggregated daily coping (frequency and flexibility) tested as mediators
Keng and Tong (2016)	395 undergraduate students (*M*_*age*_ = 22.08, 61% women); 2 days (36 signals)	6 items (1 per strategy): Active coping, reappraisal (adaptive coping), distraction, fantasising, self‐blame, venting (maladaptive coping). Instrument: Items from previous studies; 7‐point scale	None	Coping examined only as a BP mediator using aggregated indices
Marco et al. (1999)	100 adults with high levels of work or marital stress (*M*_*age*_ = 42; 50% women); 2 days (1 signal/hour)	9 items (1 per strategy): Planning, direct action (problem‐focused), distraction, situation redefinition, catharsis, acceptance, social support, relaxation, seeking spiritual support (emotion‐focused). Instrument: DCI; binary response.	Work, marital and other stressors; event stressfulness; disruptiveness, control, importance, desirability, challenge appraisals	WP MLM testing event‐level pre–post mood change as a function of appraisals and coping
Sladek et al. (2016)	71 adolescents (*M*_*age*_ = 18.85, 77% girls); 3 days (5 signals/day)	8 items (8 per coping style): Engagement coping. Instruments: Brief COPE, RSQ; 3‐point scale.	Salivary cortisol; description of the most stressful event; event stressfulness	Hierarchical linear growth model testing WP coping as a moderator of momentary stress reactivity across the day; BP trait coping predicting mean levels and cross‐level interaction
Socastro et al. (2022)	97 students (*M*_*age*_ = 20.13, 87% women); 5 days (3 signals/day)	4 items (1 per strategy): Reappraisal, active coping, avoidance, rumination. Instrument: Items from previous studies; 10‐point scale.	Event stressfulness; event controllability	MLM estimating same‐moment WP associations between stress appraisals and coping; cross‐level moderation by depression and anxiety; lagged dependent variables and day and moment covariates examined in supplementary models
Wahlers et al. (2024)	109 undergraduate students (*M*_*age*_ = 18.87, 51% women); 6 days (5 signals/day)	8 items (1 per strategy): Distraction, suppressing feelings, relaxation (emotion‐focused); planning or taking action, thinking more about the event, thinking differently about the event (problem‐focused); expressing feelings, seeking social support (mixed). Instrument: Self‐developed; binary response.	Stress level; appraisals of challenge, importance, and control; rumination	Linear MLM examining momentary stressor occurrence and negative affect, with L2 working memory capacity predicting means and moderating slopes
Wolfers et al. (2023)	218 mothers (*M*_*age*_ = 33.11); 7 days (4 signals/day)	12 items (1 per strategy): Self‐distraction, active coping, emotional social support, giving up, instrumental social support, venting, positive reappraisal, planning, humour, information seeking, taking a break, information avoidance. Instrument: Brief COPE; binary response.	Phone use in stressful situations; coping effectiveness; event controllability; event importance; urgency of responding to the event	MLM estimating momentary associations between stress and smartphone use as a coping tool; included person‐, situation‐, and device‐level predictors
Assessing coping with overall stress/stressful experiences				
Ewert et al. (2022)	213 adults (*M*_*age*_ = 23.35, 86% women); 7 days (3 signals/day)	10 items (2 per strategy): Active coping, acceptance, positive reinterpretation (engagement strategies); denial, behavioural disengagement (disengagement coping). Instrument: Brief COPE; 4‐point scale.	Stress level	Multilevel mediation models estimating same‐occasion WP indirect associations between self‐compassion and affect via perceived stress and coping; exploratory lagged effects also tested
Hirano et al. (2025)—Study 1	101 adults (*M*_*age*_ = 40.83, 52% women); 14 days (2 signals/day)	14 items (1 per strategy): Active coping, instrumental support, planning, positive reframing (problem‐focused coping); venting, emotional support, humour, acceptance, self‐blame, religion (emotion‐focused coping); self‐distraction, denial, substance use, behavioural disengagement (avoidant coping). Instrument: Brief COPE; 5‐point scale.	Stress level	Linear MLM estimating same‐day associations between daily coping and daily psychological health outcomes, with L2 self‐esteem and self‐compassion predicting average outcomes; indirect pathways via coping evaluated
Hirano et al. (2025)—Study 2	75 women (*M*_*age*_ = 41.39); 14 days (2 signals/day)	14 items (1 per strategy): Active coping, instrumental support, planning, positive reframing (problem‐focused coping); venting, emotional support, humour, acceptance, self‐blame, religion (emotion‐focused coping); self‐distraction, denial, substance use, behavioural disengagement (avoidant coping). Instrument: Brief COPE; 5‐point scale.	Stress level	Linear MLM estimating same‐day associations between daily coping and daily psychological health outcomes, with L2 self‐esteem and self‐compassion predicting average outcomes; indirect pathways via coping evaluated
Noack et al. (2025)	210 adults (*M*_*age*_ = 23.33; 86% women); 7 days (3 signals/day)	10 items (2 per strategy): Active coping (problem‐focused), positive reinterpretation, acceptance (emotional‐focused), denial, behavioural disengagement (avoidance). Instrument: Brief COPE; 4–point scale.	None	MLM testing momentary associations between self‐compassion and coping; L2 neuroticism specified as a cross‐level moderator of L1 slopes

Abbreviations: BP = between‐person; CCSC = Children's Coping Strategies Checklist; (C)ERQ = (Cognitive) Emotion Regulation Questionnaire; COPE Inventory = Coping Orientation to Problems Experienced Inventory; CSI = Coping Strategies Inventory; CTEAS = Coping Through Emotional Approach Scale; DCI = Daily Coping Inventory; EACS = Emotional Approach Coping Scale; GLMM = generalised linear mixed models; HICUPS = How I Coped Under Pressure Scale; L1 = Level 1; L2 = Level 2; MLM = multilevel modelling; MMAP = Measure of Mental Anticipatory Process; MSEM = multilevel structural equation modelling; RSQ = Responses to Stress Questionnaire; WCCL = Ways of Coping Checklist; WP = within‐person.

### 3.1 Study Design Characteristics

While 11 (20.0%) studies assessed coping multiple times daily with ESM, the majority used daily diary methods with one report per day, typically completed in the evening. Duration of daily diary studies ranged from three to 115 days, with 32 (72.7%) lasting 1–3 weeks. ESM studies were 2–14 days long and generally assessed coping 2–5 times per day, balancing the need for frequent data collection with the need to keep the study manageable for participants. Two studies (Keng and Tong 2016; Marco et al. 1999) were clear outliers, with very intensive hourly sampling, and therefore limited the protocol to 2 days.

While daily diary protocols relied on a single end‐of‐day report, ESM studies varied in how prompts were scheduled. Across the 11 ESM studies, seven used semi‐random prompts delivered within predefined time blocks. Three studies employed fixed assessment times (Hirano et al. 2025, Studies 1 and 2; Wolfers et al. 2023). One study (Sladek et al. 2016) did not send prompts; instead, participants were instructed to complete five reports per day at approximately scheduled times, immediately after providing saliva samples.

The majority of studies were conducted in student or adult samples. College and university students were represented in 23 (41.8%) studies, with mean ages between 17.8 and 23.3 years. Adults and young adults who were not part of student samples were included in 20 (36.4%) studies, with mean ages between 19.44 and 45.4 years. Children and adolescents were examined in 11 (20.0%) studies, with mean ages between 9.14 and 18.6 years. Older adults were studied in three (5.5%) studies, with mean ages between 64.7 and 74.7 years. Because two studies included mixed‐age samples, percentages across categories are not mutually exclusive.

### 3.2 Measurement of Coping Strategies

Studies varied in their approaches to measuring coping strategies, though most adapted standardized questionnaires to fit the demands of intensive longitudinal research. Researchers typically selected specific items from validated instruments and modified them for daily or momentary assessments. Commonly used instruments included the COPE Inventory (Carver et al. 1989) and the Brief COPE (Carver 1997). Half of the studies (*n* = 28; 50.1%) employed a single‐item approach to measure individual coping strategies. This approach was particularly advantageous in high‐frequency assessments such as in ESM studies, where minimising participant burden is critical. The number of coping strategies assessed also varied widely across studies. Some concentrated on a narrow selection of coping strategies of interest (e.g., Eckland and Berenbaum 2021; Galla and Wood 2015), while others included broader assessments, covering up to 14 coping strategies for a more detailed and complete analysis of coping patterns (e.g., Dunkley et al. 2014; Roesch et al., 2010a).

Across studies, the most frequently assessed coping strategies were active coping or problem solving (*n* = 37), positive reframing or cognitive restructuring (*n* = 34), avoidance, disengagement, denial or distraction (*n* = 34), seeking social or instrumental support (*n* = 28), acceptance (*n* = 26), emotional expression, venting or catharsis (*n* = 24), and planning (*n* = 21). Other fairly common strategies were religious or spiritual coping (*n* = 18), humour (*n* = 13), rumination, self‐blame, or catastrophizing (*n* = 12), relaxation (*n* = 8), and substance use (*n* = 7). In terms of broader categorisation, many researchers grouped individual coping strategies into higher‐order domains, such as problem‐focused coping, emotion‐focused coping, and avoidant coping (e.g., Hirano et al. 2025). Meanwhile, 12 (21.8%) studies did not measure coping strategies separately but instead assessed higher‐order coping styles, such as engagement versus disengagement coping (e.g., Santiago et al. 2016). Regarding response formats, 44 (80.0%) studies used Likert‐type scales, typically ranging from 4‐ to 8‐points, to measure the frequency, intensity, or extent of coping strategy use. Eleven (20.0%) studies, such as those by Blaxton and Bergeman (2017) and Friedman‐Wheeler et al. (2019), employed binary response formats (yes/no).

Most studies (*n* = 51; 92.7%) focused on retrospective coping, asking participants to reflect on the strategies they had used since their last report or since waking up. However, two studies (Neupert and Bellingtier 2019; Neupert et al. 2016) assessed anticipated coping with future stressors. Studies also varied in how they anchored coping reports to stressors. Most commonly, studies focused on a single event. Within 44 daily diary designs, 24 (43.6%) studies asked participants to report on coping with the most stressful or difficult event of the day. A smaller subset (*n* = 4) also used an event‐based approach but without explicitly requiring participants to select the most impactful one (Galla and Wood 2015; Hoggard et al. 2012; Roesch et al. 2009, 2010). In contrast, 14 (25.5%) studies did not prioritise a specific situation and instead assessed coping in relation to all stressful experiences occurring across the day. Similarly, among 11 ESM studies, four assessed coping with the most stressful event since the last prompt, while three studies (Hammond et al. 2025; Wahlers et al. 2024; Wahlers et al. 2024) used an event‐based approach but did not require participants to prioritise the most stressful event. The remaining four assessed coping in relation to all stressors or overall stress experienced within the reporting interval. The two studies that exclusively assessed anticipatory coping, conducted by Neupert and Bellingtier (2019) and Neupert et al. (2016), asked participants to evaluate how they planned to cope with each anticipated stressor separately.

In 28 (50.1%) studies, participants were instructed to either describe the stressor or select from a list of predefined daily stressors before reporting how they coped. Notably, all of these studies measured coping once per day. This approach enabled researchers to assess the perceived cause or type of stressor. For instance, Finkelstein‐Fox et al. (2022) categorised daily stressors into domains such as interpersonal conflict, academic or work‐related problems, and intrapersonal or self‐focused stress.

Among the 35 studies focussing on one stressful event, 21 studies (60.0%) also measured the perceived stressfulness of the event, and 13 studies (37.1%) assessed event controllability. For instance, Finkelstein‐Fox et al. (2019) had participants appraise each day's worst stressor for both stressfulness and controllability, treating these appraisals as within‐person variables that fluctuated daily. Coping flexibility, a concept which describes the ability to adjust coping strategies in response to varying situational demands, such as differences in how controllable or severe a stressor is (see Cheng et al. 2014), was examined in several studies. e.g., Socastro et al. (2022) examined flexibility by testing whether associations between controllability and strategy use depended on momentary stress intensity, and found that in low‐intensity stress situations, higher stress controllability was associated with greater use of reappraisal and rumination, whereas in high‐intensity stress situations, higher controllability was related to reduced use of rumination. In contrast, Hammond et al. (2025), quantified coping flexibility as within‐person variability in the use of problem‐focused, support‐seeking, and cognitive strategies across momentary stress reports, irrespective of specific situational characteristics.

### 3.3 Analytical Approaches

Across the reviewed studies, MLM was the dominant analytic framework used to examine coping in daily life, although not all studies adopted this strategy. Rather than modelling observations as nested within individuals, few studies (e.g., Hammond et al. 2025; Keng and Tong 2016; Nguyen‐Feng et al. 2017) aggregated daily coping responses to create indices such as mean frequency or variability and analysed these variables using regression or structural equation modelling. Such studies emphasise stable individual differences in typical coping patterns rather than day‐to‐day or momentary fluctuations.

Nevertheless, the large majority of studies treated repeated observations as nested within individuals, which allows researchers to separate within‐person changes from between‐person differences (for an overview of this analytical approach, see Curran and Bauer 2011). This way, researchers estimated associations among coping, stress appraisals, and psychological or behavioural outcomes at the within‐person level. However, there was substantial variation in how researchers implemented MLM with respect to temporal ordering, treatment of between‐person differences, and the modelling of mediation and moderation.

Most studies relied on concurrent associations between coping and other constructs measured within the same reporting window. In these models, coping was typically entered as a Level‐1 variable predicting affect (e.g., Blaxton and Bergeman 2017), physical symptoms (e.g., Finkelstein‐Fox et al. 2022), or behavioural outcomes (e.g., Park et al. 2004a). Even when predictors were person‐mean centred, the analyses remained cross‐sectional. A smaller but notable subset of studies incorporated lagged or prospective structures, in which usually coping at one occasion predicted outcomes at a later time point. Examples include prior‐day coping predicting next‐morning anxiety (Iida et al. 2017), evening coping predicting subsequent sleep (Yap et al. 2021), or coping mediating links between mindfulness and later vitality across measurement occasions (Wu and Buchanan 2019). In some investigations, lagged effects were central to the research question (e.g., Iida et al. 2017), whereas in others they complemented primary concurrent analyses (e.g., Ewert et al. 2022).

Cross‐level interactions were frequently used to evaluate whether stable characteristics shaped day‐to‐day coping processes. Traits such as neuroticism (Gunthert et al. 1999), ethnicity or cultural variables (Sasaki and Kim 2011; Santiago et al. 2016), self‐control (Galla and Wood 2015), or victimization history (Finkelstein‐Fox et al. 2022) frequently predicted average levels of coping, and in many cases, moderated within‐person associations.

Coping was also regularly examined as a potential mediating mechanism. Two general strategies appeared. The first involved within‐person concurrent mediation, in which variables assessed at the same occasion were arranged into indirect pathways (e.g., Dunkley et al. 2014; Finkelstein‐Fox et al. 2022; Monroy et al. 2021; Weinstein et al. 2009). The second involved between‐person mediation, where coping was aggregated across days and used to explain links between baseline predictors and later outcomes (e.g., Finkelstein‐Fox et al. 2019; Nguyen‐Feng et al. 2017).

Explicit modelling of developmental change in coping was uncommon. An exception is Valiente et al. (2015), who used latent growth curve modelling across multiple diary bursts spanning several years to estimate mean‐level change and individual differences in trajectories across adolescence.

Finally, information about statistical power or sample‐size justification was inconsistently reported. Seventeen (30.9%) studies provided some indication of how sample size decisions were made. Nine studies reported ad‐hoc power analyses, four based sample sizes on prior literature or methodological recommendations, and four conducted post‐hoc calculations. Many studies did not provide additional information regarding how sample size was determined in relation to the complexity of the multilevel models estimated.

## 4 Discussion

This review synthesised 55 intensive longitudinal studies using daily diary and ESM to investigate coping strategies. By allowing participants to report in real time or at the end of the day, these methods reduce recall bias and capture nuances that traditional retrospective surveys often miss (Bolger et al. 2003; Litt et al. 2011). Our goals were to characterise how included studies were designed, how coping was measured, and how dynamic data were analysed. Bringing these strands together reveals a field that has made major methodological progress, yet still faces persistent conceptual and analytical challenges in capturing coping as a situational, unfolding process.

### 4.1 Design Considerations

The reviewed studies reveal a clear dominance of pragmatic and feasible study designs. Most researchers relied on once‐per‐day assessments administered over one to 3 weeks, whereas high‐frequency sampling was comparatively rare. Event‐contingent approaches were not represented among the included studies. However, this absence should be interpreted in light of our eligibility criteria. Because the review focused on studies assessing coping with naturally occurring everyday stressors using repeated daily diary or ESM assessments, event‐contingent designs—particularly those targeting specific types of stressful events—were less likely to be represented in the present review. Consequently, the absence of such designs does not necessarily indicate that they are uncommon in the broader coping literature. Within the body of studies included in this review, researchers generally prioritised protocols that participants can realistically maintain in everyday context, reflecting the trade‐off between ecological validity and participant burden. Dense sampling can capture short‐term dynamics, but it also increases the risk of fatigue, lower compliance, and selective dropout (Conner and Lehman 2012; Scollon et al. 2003). In contrast, end‐of‐day reports are easier for participants to maintain but may miss rapid changes in appraisals and strategy use that occur within the day. As a result, our understanding of coping in everyday life is partly shaped by what can realistically be assessed in natural settings.

Nevertheless, event‐contingent sampling remains an important methodological option, particularly when the aim is to study coping with discrete or relatively infrequent stressors. Compared with signal‐contingent approaches, it may better capture events that occur outside scheduled assessment windows, although it also places greater demands on participants and may not be practical in many everyday situations (Scollon et al. 2003). Regardless of the sampling strategy, retrospective reporting—whether collected shortly after an event or in end‐of‐day summaries—remains susceptible to recall bias (Larson and Csikszentmihalyi 2014). Beyond considerations of feasibility, the choice of sampling schedule also has theoretical implications. Sampling schedules should follow from explicit assumptions about when coping processes are expected to unfold. Brown and Stenling (2025) argue that mismatches between hypothesised time scales and measurement intervals may result in studies that are unable to address their intended questions. If coping responses are theorized to influence outcomes over hours, daily assessments may blur these dynamics; conversely, very dense sampling may increase burden without yielding additional insight. Making these assumptions explicit can therefore strengthen the alignment between theory and design.

To better capture coping in everyday life, future studies may consider combining complementary assessment strategies. For example, end‐of‐day summaries could be used alongside signal‐contingent assessments to capture stressors occurring outside scheduled prompts. Importantly, when participants are asked to reflect on how they coped with ‘stressors in general’, researchers should be cautious, as such aggregated reports lose situational specificity and obscure how coping strategies relate to stressor features like controllability or intensity (Todd et al. 2004).

### 4.2 Measurement Issues and Recommendations

Efforts to reduce participant burden also affected coping measures. To make repeated assessments feasible, most researchers shortened traditional scales or used single items. While this helps reduce participant fatigue and improve compliance, it can also come at the cost of reliability or depth (Horstmann 2021), and limits comparability across studies. To deal with this trade‐off, Hulin et al. (2024) have used item sampling, where only a subset of items is asked at each prompt. This approach can include rotating items across measurement points or spreading multi‐item scales over several days. As Horstmann (2021) noted, strategies like item sampling, planned missingness, and measurement bursts can help balance feasibility with data quality. Planned missingness introduces intentional gaps in the data that still allow for valid inferences across participants, while measurement bursts involve collecting dense data over short periods to capture dynamic processes without overloading participants.

A particularly encouraging observation of this review is the frequent inclusion of stressor appraisals. Several studies incorporated at least perceived intensity or controllability, which makes it possible to examine whether individuals match their coping strategies to situational demands— often referred to as coping flexibility, or the ability to shift strategies depending on the situation (Cheng et al. 2014). This perspective shifts the focus from asking which strategy is best towards examining when a particular strategy is most useful. In doing so, it aligns with the goodness‐of‐fit hypothesis, which posits that coping is most effective when it aligns with the stressor's demands (Lazarus and Folkman 1984).

From a measurement standpoint, this implies that coping cannot be evaluated without reference to the situations in which it occurs. Repeated‐measures studies have shown that individuals adjust their strategies depending on how they appraise each stressor. On more controllable days, people tend to use problem‐focused coping; under less controllable conditions, they shift towards acceptance, distraction, or emotion‐focused strategies (Cheng et al. 2014; Dunkley et al. 2014; Finkelstein‐Fox et al. 2022; Socastro et al. 2022; Litt et al. 2011). Without assessing these appraisals, such context‐dependent patterns would remain invisible. Importantly, situational context also includes the temporal characteristics of the stressor. Stressors differ in whether they can be anticipated, whether they are brief or prolonged, whether they constitute discrete events or ongoing difficulties, etc. These characteristics may shape which coping responses are possible and when they can meaningfully be assessed. e.g., anticipatory coping presupposes a foreseeable stressor, whereas coping with a long‐lasting problem may involve strategies that change as the stressor unfolds. Studies included in this review demonstrate that such characteristics can be incorporated into daily‐life measurement: Stone et al. (1995) assessed both degree of stressor anticipation and whether the reported problem was a single event or long‐lasting, while Neupert and Bellingtier (2019) and Neupert et al. (2016) explicitly assessed anticipatory coping with expected future stressors. The implication is that measurement approaches focussing solely on the frequency of strategy use risk oversimplifying coping processes. The emotional consequences of coping depend not only on what individuals do but also on stressor characteristics, available resources, and broader contextual factors (e.g., Iida et al. 2017). Capturing coping adequately therefore requires instruments that embed responses within situational information.

These considerations also help explain why coping remains underrepresented in intensive longitudinal research compared to emotion regulation (Trudel‐Fitzgerald et al. 2024). This difference may partly come from their conceptual scope. Emotion regulation refers to the modulation of emotional states regardless of external triggers (Gross 2024), while coping is a broader process involving emotional, cognitive, and behavioural responses to specific external stressors (Lazarus and Folkman 1984). From the measurement perspective, this distinction is critical. Emotion regulation can be measured with brief, decontextualised items suited for high‐frequency sampling. Coping, which depends on stressor‐specific context and often unfolds over longer periods, is harder to capture in real time. As a result, coping assessments in diary and ESM research may be less frequent, more heterogeneous, or reduced in complexity (Bolger et al. 2003; Trudel‐Fitzgerald et al. 2024). Moreover, substantial overlap exists between the two traditions. Strategies such as reappraisal or seeking support are sometimes labelled as emotion regulation and sometimes as coping. For measurement, this creates challenges for comparability across studies. To address this, researchers should adopt unified, function‐based taxonomies that characterise strategies based on their temporal position relative to stressor (e.g., anticipatory vs. reactive), target (e.g., internal emotional states vs. external stressors), and function (e.g., reducing distress, solving a problem, altering the meaning of the situation). Importantly, such a taxonomy does not imply that the same measurement approach is appropriate for all daily stressors. Rather, the specific coping processes assessed should reflect the characteristics of the stressors under investigation and the research question. Thus, a common conceptual framework could improve comparability across studies while retaining the flexibility needed to capture coping with heterogeneous daily stressors (Gross 2024; Trudel‐Fitzgerald et al. 2024).

### 4.3 Analytical Practices and Future Directions

Beyond questions of design and measurement, the reviewed studies also differed in how intensive longitudinal data were analysed. Multilevel modelling has become the default analytic approach, which is a major strength because it distinguishes within‐person changes from between‐person differences (Curran and Bauer 2011). This distinction is central to coping theories that emphasise contextual adaptation (Lazarus and Folkman 1984). e.g., Aldridge and Roesch (2008) showed that adolescents who generally relied on acceptance reported better overall adjustment, yet on days when they used more acceptance than usual, they experienced both higher positive and negative affect. Such patterns are visible only when within‐person variation is modelled explicitly.

At the same time, most analyses were concurrent. Even when person‐mean centring was applied, coping and outcomes were typically measured within the same reporting window, limiting conclusions to co‐occurrence rather than temporal order. Consequently, we still know relatively little about how coping efforts accumulate, fade, or shape later experiences. When lagged models were applied, they provided valuable process‐oriented insights, suggesting that some strategies may show delayed well‐being or health benefits, whereas others carry short‐term costs (e.g., Ward et al. 2023; Yap et al. 2021).

Researchers have also begun integrating mediation and moderation into intensive longitudinal designs to address when and for whom coping is effective (e.g., Finkelstein‐Fox et al. 2022; Sasaki and Kim 2011). Yet many models relied on same‐day data, limiting temporal interpretations. Moving forward, greater use of temporally explicit models, such as dynamic structural equation models (McNeish and Hamaker 2020), will be necessary to clarify whether coping processes mediate or moderate stress‐related outcomes in a temporally meaningful way.

Complementing these approaches, nonlinear analytic frameworks such as dynamical systems approaches may provide additional insights by conceptualising coping as a dynamic process unfolding over time and capturing patterns of adaptation that may not be fully represented by conventional linear models (Hill et al. 2025).

These methodological advances also have important implications for study design. Different analytic goals place different demands on the data, including requirements related to statistical power (Snijders 2005). Within‐person associations benefit from many repeated observations, whereas between‐person effects and cross‐level interactions depend primarily on the number of participants. Lagged hypotheses are especially demanding because they require both sufficient observations and measurement intervals that match the theorized process (Brown and Stenling 2025).

Although some authors reported ad‐hoc or post‐hoc power analyses or relied on precedents from earlier studies, explicit links between sample size and model complexity were not always provided. Simulation methods such as Monte Carlo can support planning by evaluating whether a proposed combination of subjects and observations is likely to detect plausible effects (Muthén and Muthén 2002; Schultzberg and Muthén 2018). After data collection, Bayesian multilevel modelling provides a complementary option. The accumulating diary and EMA literature can inform reasonable prior expectations, which may improve estimation precision, particularly in complex models or when Level‐2 samples are modest (Dora et al. 2024). These approaches cannot compensate for fundamentally insufficient data, but they can help researchers state their assumptions clearly and plan studies in a more realistic way.

### 4.4 Limitations of This Review

This review has several limitations. First, to avoid disproportionate representation of the same dataset and study design, we included only one article when multiple publications were based on the same sample. This decision was consistent with the methodological focus of the review, which aimed to summarise how coping has been assessed in intensive longitudinal research. Nevertheless, different papers based on the same dataset may apply different analytic approaches or emphasise different methodological aspects. Consequently, some methodological variability—particularly with respect to analytic approaches—may be underrepresented in the present synthesis. Second, we restricted inclusion to studies examining coping with naturally occurring daily stressors and excluded those centred on predefined domains such as discrimination, academic or work‐related stress, in which participants were asked to report only on that specific type of event rather than on any stressor they experienced. This decision allowed us to examine coping across a broad range of everyday situations. However, it also means that our conclusions may not fully generalise to contexts in which the type of stressor is restricted. Because studies focussing on specific categories of stressors may be more likely to use event‐contingent designs, such methodological approaches may also be underrepresented in the present synthesis. Third, grouping coping strategies and design features required interpretive judgements. Studies did not always use identical terminology or definitions, and some strategies could reasonably fit into more than one category. As a result, other reviewers might have organised certain elements differently. These challenges were particularly pronounced when classifying analytic approaches, where overlapping terminology and variations in model specification often made clear distinctions difficult.

## 5 Conclusion

Intensive longitudinal research has reshaped how we understand coping, highlighting its dynamic, situational nature in everyday stress experiences. The field now has strong tools for detecting within‐person variability and situational fit. The next challenge is to move beyond documenting associations towards modelling temporal processes, clarifying mechanisms, and aligning measurement schedules with theoretical expectations. Meeting these goals will allow future research to more fully capture coping as it is lived: dynamic, contextual, and continuously evolving.

## Conflicts of Interest

The authors declare no conflicts of interest.

## Data Availability

Data sharing not applicable to this article as no datasets were generated or analysed during the current study.
